# Substituted *N*-Phenylpyrazine-2-carboxamides: Synthesis and Antimycobacterial Evaluation

**DOI:** 10.3390/molecules14104180

**Published:** 2009-10-20

**Authors:** Martin Doležal, Jan Zitko, Diana Kešetovičová, Jiří Kuneš, Michaela Svobodová

**Affiliations:** 1 Faculty of Pharmacy in Hradec Králové, Charles University in Prague, Heyrovského 1203, Hradec Králové, 500 05, Czech Republic; E-Mails: jan.zitko@faf.cuni.cz (J.Z.); diana.kesetovicova@faf.cuni.cz (D.K.); jiri.kunes@faf.cuni.cz (J.K.); 2 Department of Microbiology, Regional Hospital, Kyjevská 44, Pardubice, 532 03, Czech Republic; E-Mail: michaela.svobodova@nemocnice-pardubice.cz (M.S.)

**Keywords:** pyrazinecarboxamide synthesis, *in vitro* antimycobacterial screening, lipophilicity, SAR

## Abstract

The condensation of chlorides of substituted pyrazinecarboxylic acids with ring-substituted anilines yielded twelve substituted pyrazinecarboxylic acid amides. The synthetic approach, analytical, and lipophilicity data of the newly synthesized compounds are presented. Two antituberculosis assays were used. Firstly, the antimycobacterial activity against four different *Mycobacterium* strains in a series of pyrazine derivatives was investigated. Secondly, the antimycobacterial evaluation was performed at the Tuberculosis Antimicrobial Acquisition and Coordinating Facility (TAACF) program. Interesting *in vitro* antimycobacterial activity was found, *N*-(3-iodo-4-methyl-phenyl)pyrazine-2-carboxamide (**9**) was most active derivative compound against *M. tuberculosis* (MIC < 2.0 μmol/L), while another iodo derivative 5-*tert*-butyl-6-chloro-*N*-(3-iodo-4-methyl-phenyl)pyrazine-2-carboxamide (**12**) was the most active compound in the TAACF antituberculosis screening program (IC_90_ = 0.819 µg/mL).

## 1. Introduction

Every year, tuberculosis (TB) kills some 3.1 million people, more deaths than those caused by any other single bacterial disease. The World Health Organization has expressed concern over the emergence of virulent drug-resistant strains of TB and is calling for measures to be strengthened and implemented to prevent the global spread of these deadly TB strains. Multidrug Resistant TB (MDR-TB) describes strains of tuberculosis that are resistant to at least the two main first-line TB drugs—isoniazid (INH) and rifampicin. Extensive Drug Resistant TB (also referred to as Extreme Drug Resistance, XDR-TB) is MDR-TB that is also resistant to three or more of the six classes of second-line drugs. This follows research showing the extent of XDR-TB, a newly identified TB threat which leaves patients (including many people living with HIV) virtually untreatable using currently available anti-TB drugs. An urgent need is to develop new agents active against resistant bacteria, and having different mechanism of action [1,2].

Pyrazine derivatives are important drugs with antibacterial, diuretic, hypolipidemic, antidiabetic, hypnotic, anticancer and antiviral activity. Pyrazinamide (PZA), another first-line TB drug, was discovered through an effort to find antitubercular nicotinamide derivatives [3]. The activity of PZA appears to be pH dependent, since it is bactericidal at pH 5.5, but inactive at neutral pH. PZA is used in combinations with INH and rifampicin. It is especially effective against semi-dormant mycobacteria. Its mechanism of action appears to involve its hydrolysis to pyrazinoic acid via the bacterial enzyme pmcA [4]. PZA can also be metabolized by hepatic microsomal deamidase to pyrazinoic acid, which is a substrate for xanthine oxidase, affording 5-hydroxypyrazinoic acid. The acid is believed to act as an antimetabolite of nicotinamide and interferes with NAD biosynthesis. Pyrazinoic acid disrupts membrane energetics and inhibits membrane transport function in *M. tuberculosis* [5]. The resistance on PZA arises by the absence of the enzyme, Pmc A. The major side effect of PZA is a dose-related hepatotoxicity. A different analog of PZA, 5-chloropyrazine-2-carboxamide, has previously been shown to inhibit mycobacterial fatty acid synthase I (FAS I) [6].

Our research is focused on PZA analogues with a -CONH- bridge connecting the pyrazine and benzene rings. This moiety can form centrosymmetric dimer pairs with the peptidic carboxamido group of some peptides, needed for binding to the receptor site, possibly by formation of hydrogen bonds. These pyrazine derivatives were previously studied for their herbicidal effects [7,8,9]. All substituted amides of pyrazinecarboxylic acid studied can be interpreted as more lipophilic aza-analogues of nicotinamide [7,8,9,10,11]. Substituted quinoxalinecarboxylic acid amides (with a visible PZA fragment in a molecule) were identified as the active antimycobacterial derivatives with Minimal Inhibition Concentrations (MICs) on the same order as rifampicin [12]. The report that 5-chloropyrazine-2-carboxamide has a different mode of action than PZA itself makes this research direction more than relevant [13].

**Figure 1:**
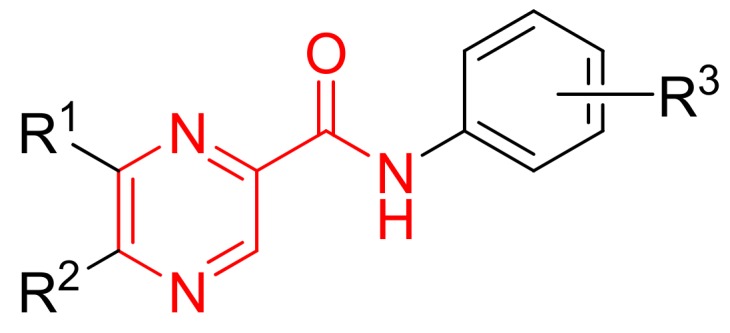
Pyrazinamide structure modification (see the pyrazinamide fragment, red colour.

This study deals with the PZA structure modification and is based on rich substitution both in the pyrazine (R^1^ and R^2^) and benzene (R^3^) parts of the target molecule (see Figure 1). The aim of this work is to find the structure-activity relationship (SAR) in the mentioned series, *i.e.* to continue in the study of the substituent variability influence on the antimycobacterial activity, and to determine the importance of increased lipophilic properties for biological effect of the newly prepared substituted pyrazinecarboxamides.

## 2. Results and Discussion

**Scheme 1:**
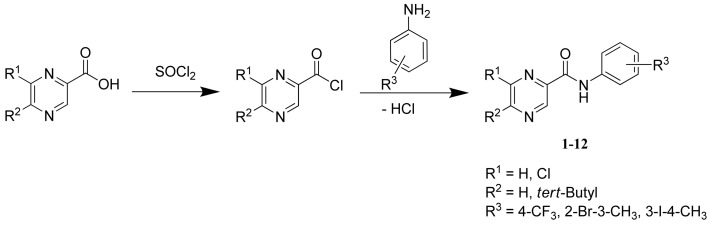
Synthesis and structure of prepared compounds **1**-**12**.

An approach that had been chosen to develop such compounds is structure analogy with PZA, a first-line agent in treatment of tuberculosis. This paper deals with SAR of some PZA analogues derived from binuclear compounds with the -CONH- linker (see Figure 1 and Scheme 1).

The first step of the study was chemical synthesis of new analogues. The condensation of chlorides of substituted pyrazinecarboxylic acids with ring-substituted anilines yielded twelve substituted pyrazinecarboxylic acid amides (see Scheme 1). We chose quite hydrophobic electron-withdrawing (halogens), and bulky (*tert*-butyl) substitutents on the pyrazine or benzene part. Chemical synthesis was followed by structure confirmation and determination of lipophilicity (log *P*, Clog *P* calculations).

### 2.1. Results of antimycobacterial screening

Biological analysis comprised antimycobacterial activity screening as a main task. Two antituberculosis assays were used for antimycobacterial evaluation of prepared compounds **1**-**12**. The MIC estimation against four different *Mycobacterium* strains in a series of pyrazine derivatives was the first one investigated. Mycobacteria other than tuberculosis (MOTT) are mycobacterial species that may cause human diseases but do not cause tuberculosis. Several of prepared compounds exhibited relatively high antimycobacterial activity against *M. tuberculosis*, namely *N*-(4-trifluoro-methylphenyl)pyrazine-2-carboxamide (**1**), *N*-(2-bromo-3-methylphenyl)pyrazine-2-carboxamide (**5**), and *N*-(3-iodo-4-methylphenyl)pyrazine-2-carboxamide (**9**). These structures exhibited Minimal Inhibition Concentrations (MICs) ≤ 2 mg/L. The susceptibility against several MOTTs strains (*M. kansasii*, *M. avium*) was very low or negative in our series. Obtained results give a good view onto SAR of these analogues and promise even better activity after some structure optimization experiments. A second antimycobacterial evaluation was performed at the Tuberculosis Antimicrobial Acquisition and Coordinating Facility (TAACF) program. 5-*tert*-Butyl-6-chloro-*N*-(3-iodo-4-methylphenyl)pyrazine-2-carboxamide (**12**) was the most active compound at the TAACF antituberculosis screening (IC_90_ = 0.819 µg/mL). From the SAR point of view the importance of iodine substitution in position 3 of benzene ring for the antimycobacterial activity was identified, mostly in compounds **9** and **12**. The discrepancy between the results of two antimycobacterial assays can be explained by the using of different laboratory conditions (pH, growth medium). Acidic pH (pH = 5.5) is crucial for the mode of action of PZA where PZA as a prodrug is converted into active form of pyrazinoic acid inside the bacilli [14]. For the results see Table 1.

**Figure 2:**
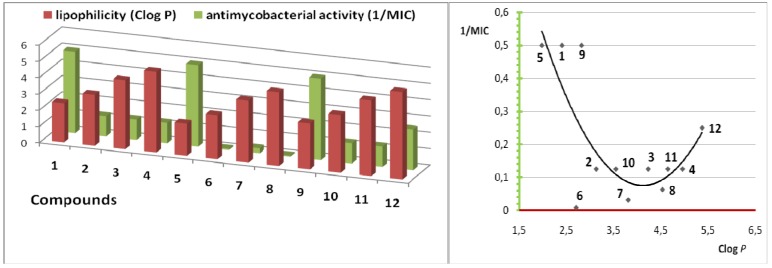
Antimycobacterial activity (against *M. tuberculosis*, 1/MIC) vs. lipophilicity (Clog *P*) in compounds **1**-**12**.

The drugs cross biological barriers most frequently through passive transport, which strongly depends on their lipophilicity. Lipophilicity (Clog *P*) has been studied in the realationship to the antimycobacterial activity (1/MIC) in series of compounds **1**-**12**, see Figure 2. The optimal Clog *P* values for the antimycobacterial activity may not cross the 3.0 or must be higher than 5.3 value in our series; this observation is in good agreement with our previous results [11].

In general, the less active compounds may still have significant inhibitory activity, and this data should not be ignored; their analogues, derivatives, and alterations in physical properties may confer significant changes in biological effects. Therefore, synthesis and evaluation of other pyrazinecarboxylic acid derivatives are necessary to broaden the structure-activity data.

## 3. Experimental Section

### 3.1. Chemistry

#### 3.1.1. Instrumentation and chemicals

All organic solvents used for the synthesis were of analytical grade. The solvents were dried and freshly distilled under argon atmospheres. Melting points were determined using a SMP 3 Melting Point Apparatus (BIBBY Stuart Scientific, UK) and are uncorrected. The reactions were monitored and the purity of the products was checked by TLC (Merck UV 254 TLC plates, Darmstadt, Germany) using petroleum ether/EtOAc (9:1).as developing solvent. Purification of compounds was made using Flash Master Personal chromatography system from Argonaut Chromatography (Argonaut Technologies, Redwood City, CA, USA) with gradient of elution from 0% to 20% ethyl-acetate in hexane. As sorbent, Merck Silica Gel 60 (0.040–0.063 mm) was used (Merck). Elemental analyses were performed on an automatic microanalyser CHNS-O CE instrument (FISONS EA 1110, Milano, Italy). Infrared spectra were recorded in Nicolet Impact 400 spectrometer in KBr pellets. ^1^H- and ^13^C- NMR Spectra were recorded (at 300 MHz for ^1^H and 75 MHz for ^13^C) in CDCl_3_ solutions at ambient temperature on a Varian Mercury—Vx BB 300 instrument (Varian, Palo Alto CA, USA). The chemical shifts were recorded as δ values in ppm and were indirectly referenced to tetramethylsilane (TMS) *via* the solvent signal (7.26 for ^1^H and 77.0 for ^13^C in CDCl_3_).

#### 3.1.2. General procedure for the synthesis of compounds **1**-**12**

A mixture of acid, *i.e.,* pyrazine-2-carboxylic, 6-chloropyrazine-2-carboxylic [15], 5-*tert*-butylpyrazine-2-carboxylic [7] or 5-*tert-*butyl-6-chloropyrazine-2-carboxylic [7] acid, respectively, (50.0 mmol) and thionyl chloride (5.5 mL, 75.0 mmol) in dry toluene (20 mL) was refluxed for about 1 h. Excess thionyl chloride was removed by repeated evaporation with dry toluene *in vacuo*. The crude acyl chloride dissolved in dry acetone (50 mL) was added dropwise to a stirred solution of the corresponding substituted amine (50.0 mmol) and pyridine (50.0 mmol) in dry acetone (50 mL) kept at room temperature. After the addition was complete, stirring was continued for 30 min, then the reaction mixture was poured into cold water (100 mL) and the crude amide was collected and purified by the column chromatography.

*N-(4-Trifluoromethylphenyl)pyrazine-2-carboxamide* (**1**). Yield 71%; Anal. Calcd. for C_12_H_8_F_3_N_3_O (267.2): 53.94% C, 3.02% H, 15.73% N; Found: 54.08% C, 3.02% H, 15.98% N; Mp 176.0–177.5 °C; Log *P*: 1.51; Clog *P*: 2.40270; IR: 3345, 3086, 1683, 1598, 1532, 1322, 1164, 1117, and 1069 cm^-1^; ^1^H-NMR (CDCl_3_) δ: 9.82 (1H, bs, NH), 9.52 (1H, d, *J* = 1.1 Hz, H3), 8.84 (1H, d, *J* = 2.5 Hz, H6), 8.63-8.60 (1H, m, H5), 7.93–7.85 (2H, m, AA´, BB´, H3´, H5´), and 7.70–7.62 (2H, m, AA´, BB´, H2´, H6´); ^13^C-NMR (CDCl_3_) δ: 160.9, 147.9, 144.8, 143.8, 142.4, 140.2, 126.4 (q, *J* = 3.7 Hz), 126.3 (q, *J* = 31.5 Hz), 124.0 (q, *J* = 271.4 Hz), and 119.5.

*6-Chloro-N-(4-trifluoromethylphenyl)pyrazine-2-carboxamide* (**2**). Yield 86%; Anal. Calcd. for C_12_H_7_ClF_3_N_3_O (301.7): 47.78% C, 2.34% H, 13.96% N; Found: 48.07% C, 2.37% H, 13.97% N; Mp 127.4–128.0 °C; Log *P*: 2.41; Clog *P*: 3.12836; IR: 3366, 3050, 1700, 1598, 1537, 1322, 1173, 1120, and 1065 cm^-1^; ^1^H-NMR (CDCl_3_) δ: 9.54 (1H, bs, NH), 9.41 (1H, s, H3), 8.84 (1H, s, H5), 7.94–7.86 (2H, m, AA´, BB´, H3´, H5´), and 7.71–7.62 (2H, m, AA´, BB´, H2´, H6´); ^13^C-NMR (CDCl_3_) δ: 159.7, 148.0, 147.5, 143.4, 142.3, 139.8, 126.5 (q, *J* = 4.0 Hz), 126.4 (q, *J* = 32.9 Hz), 123.9 (q, *J* = 264.5 Hz), and 119.7. 

*5*-*tert-Butyl-N-(4-trifluoromethylphenyl)pyrazine-2-carboxamide* (**3**). Yield 57%; Anal. Calcd. for C_16_H_16_F_3_N_3_O (323.3): 59.44% C, 4.99% H, 13.00% N; Found: 59.54% C, 3.97% H, 12.94% N; Mp 117.2–118.0 °C; Log *P*: 3.64; Clog *P*: 4.22870; IR: 3342, 2972, 2939, 2911, 2874, 1686 (CO), 1597, 1530, 1325, 1160, 1125, and 1067 cm^-1^; ^1^H-NMR (CDCl_3_) δ: 9.81 (1H, bs, NH), 9.40 (1H, d, *J* = 1.5 Hz, H3), 8.64 (1H, d, *J* = 1.5 Hz, H6), 7.93–7.85 (2H, m, AA´, BB´, H3´, H5´), 7.69–7.61 (2H, m, AA´, BB´, H2´, H6´), and 1.45 (9H, s, CH_3_). ^13^C-NMR (CDCl_3_) δ: 165.1, 160.1, 145.9, 140.6, 140.4, 140.1, 126.4 (q, *J* = 3.8 Hz), 126.0 (q, *J* = 32.7 Hz), 124.0 (q, *J* = 271.7 Hz), 119.5, 39.1, and 28.2.

*5-tert-Butyl-6-chloro-N-(4-trifluoromethylphenyl)pyrazine-2-carboxamide* (**4**). Yield 67%; Log *P*: 4.54; Clog *P*: 4.95436. Analytical data of compound **4** are consistent with the literature data [9]. 

*N-(2-Bromo-3-methylphenyl)pyrazine-2-carboxamide* (**5**). Yield 75%; Anal. Calcd. for C_12_H_10_BrN_3_O (291.1): 49.34% C, 3.45% H, 14.38% N; Found: 49.20% C, 3.48% H, 14.18% N; Mp 166.4–167.0 °C; Log *P*: 1.90; Clog *P*: 1.97804; IR: 3315, 3065, 1695, 1597, 1538, 1392, 1171, 1137, and 1020 cm^-1^; ^1^H-NMR (CDCl_3_) δ: 10.45 (1H, bs, NH), 9.51 (1H, d, *J* = 1.6 Hz, H3), 8.82 (1H, d, *J* = 2.5 Hz, H6), 8.65 (1H, dd, *J* = 2.5 Hz, *J* = 1.6 Hz, H5), 8.44 (1H, dd, *J* = 8.2 Hz, *J* = 1.1 Hz, H6´), 8.28 (1H, t, *J* = 8.0 Hz, H5´), 7.09–7.04 (1H, m, H4´), and 2.46 (3H, s, CH_3_); ^13^C-NMR (CDCl_3_) δ: 160.8, 147.6, 144.7, 144.5, 142.6, 138.7, 135.4, 127.7, 126.6, 118.9, 116.7, and 23.8.

*6-Chloro-N-(2-bromo-3-methylphenyl)pyrazine-2-carboxamide* (**6**). Yield 83%; Anal. Calcd. for C_12_H_9_BrClN_3_O (326.6): 44.13% C, 2.78% H, 12.87% N; Found: 44.22% C, 2.79% H, 12.68% N; Mp 139.0–140.0 °C; Log *P*: 2.81; Clog *P*: 2.70369; IR: 3316, 3053, 1692, 1599, 1538, 1382, 1170, 1127, and 1015 cm^-1^; ^1^H-NMR (CDCl_3_), δ: 10.21 (1H, bs, NH), 9.39 (1H, s, H3), 8.82 (1H, s, H5), 8.38 (1H, d, *J* = 8.0 Hz, H6´), 7.28 (1H, t, *J* = 8.0 Hz, H5´), 7.11–7.05 (1H, m, H4´), and 2.47 (3H, s, CH_3_); ^13^C-NMR (CDCl_3_) δ: 159.5, 147.7, 147.6, 144.0, 142.1, 138.8, 135.0, 127.7, 126.9, 119.0, 116.9, and 23.8.

*5-tert-Butyl-N-(2-bromo-3-methylphenyl)pyrazine-2-carboxamide* (**7**). Yield 89%; Anal. Calcd. for C_16_H_18_BrN_3_O (348.1): 55.18% C, 5.21% H, 12.07% N; Found: 55.30% C, 5.32% H, 12.05% N; Mp 117.3–118.5 °C; Log *P*: 4.03; Clog *P*: 3.80404; IR: 3318, 2959, 2931, 2904, 2868, 1698, 1596, 1530, 1397, 1144, and 1026 cm^-1^; ^1^H-NMR (CDCl_3_) δ: 10.45 (1H, bs, NH), 9.40 (1H, d, *J* = 1.5 Hz, H3), 8.71 (1H, d, *J* = 1.5 Hz, H6), 8.45 (1H, dd, *J* = 8.0 Hz, *J* = 1.1 Hz, H6´), 7.27 (1H, t, *J* = 8.0 Hz, H5´), 7.08–7.02 (1H, m, H4´), 2.46 (3H, s, CH_3_), and 1.46 (9H, s, CH_3_); ^13^C-NMR (CDCl_3_) δ: 167.8, 161.3, 142.9, 141.6, 139.4, 128.6, 135.6, 127.6, 126.3, 118.8, 116.6, 37.1, 29.7 and 23.8.

*5-tert-Butyl-6-chloro-N-(2-bromo-3-methylphenyl)pyrazine-2-carboxamide* (**8**). Yield 86%; Anal. Calcd. for C_16_H_17_BrClN_3_O (382.7): 50.22% C, 4.48% H, 10.98% N; Found: 50.12% C, 4.59% H, 10.92% N; Mp 145.1–146.2 °C. Log *P*: 4.93; Clog *P*: 4.52969; IR: 3316, 2992, 2978, 2955, 2930, 2871, 1702, 1596, 1526, 1396, 1148, and 1060 cm^-1^; ^1^H-NMR (CDCl_3_), δ: 10.21 (1H, bs, NH), 9.26 (1H, s, H3), 8.40 (1H, dd, *J* = 8.0 Hz, *J* = 1.1 Hz, H6´), 7.27 (1H, t, *J* = 8.0 Hz, H5´), 7.09–7.04 (1H, m, H4´), 2.47 (3H, s, CH_3_), and 1.56 (9H, s, CH_3_); ^13^C-NMR (CDCl_3_) δ: 164.7, 160.0, 145.9, 141.2, 140.1, 138.8, 135.3, 127.6, 126.6, 118.9, 116.8, 39.0, 28.3, and 23.8.

*N-(3-Iodo-4-methylphenyl)pyrazine-2-carboxamide* (**9**). Yield 84%; Anal. Calcd. for C_12_H_10_IN_3_O (339.1): 42.50% C, 2.97% H, 12.39% N; Found: 42.79% C, 3.07% H, 12.10% N; Mp 142.4–143.5 °C; Log *P*: 2.43; Clog *P*: 2.81804; IR: 3344, 3072, 1693, 1597, 1533, 1372, 1170, 1123, and 1020 cm^-1^; ^1^H-NMR (CDCl_3_), δ: 9.59 (1H, bs, NH), 9.49 (1H, d, *J* = 1.4 Hz, H3), 8.81 (1H, d, *J* = 2.5 Hz, H6), 8.58 (1H, dd, *J* = 2.5 Hz, *J* = 1.4 Hz, H5), 8.22 (1H, d, *J* = 2.2 Hz, H2´), 7.68 (1H, dd, *J* = 8.2 Hz, *J* = 2.2 Hz, H6´), 7.24 (1H, d, *J* = 8.2 Hz, H5´), and 2.42 (3H, s, CH_3_); ^13^C-NMR (CDCl_3_) δ: 160.5, 147.6, 144.6, 111.1, 142.3, 137.9, 135.7, 129.7, 129.7, 119.6, 100.8 and 27.4.

*6-Chloro-N-(3-iodo-4-methylphenyl)pyrazine-2-carboxamide* (**10**). Yield 83%; Log *P*: 3.33; Clog *P*: 3.54369. Analytical data of compound **10** are consistent with the literature data [9].

*5-tert-Butyl-N-(3-iodo-4-methylphenyl)pyrazine-2-carboxamide* (**11**). Yield 88%. Anal. Calcd. for C_16_H_18_IN_3_O (395.3): 48.62% C, 4.59% H, 10.63% N; Found: 48.61% C, 4.67% H, 10.56% N; Mp 158.4–159.7 °C. Log *P*: 4.56; Clog *P*: 4.64404; IR: 3345, 2963, 2934, 2908, 2869, 1688, 1583, 1519, 1387, 1141, and 1030 cm^-1^; ^1^H-NMR (CDCl_3_) δ: 9.57 (1H, bs, NH), 9.38 (1H, d, *J* = 1.5 Hz, H3), 8.60 (1H, d, *J* = 1.5 Hz, H6), 8.23 (1H, d, *J* = 2.2 Hz, H2´), 7.67 (1H, dd, *J* = 8.2 Hz, *J* = 2.2 Hz, H6´), 7.23 (1H, d, *J* = 8.2 Hz, H5´), 2.41 (3H, s, CH_3_), and 1.45 (9H, s, CH_3_); ^13^C-NMR (CDCl_3_) δ: 167.9, 161.0, 143.0, 141.1, 139.0, 137.6, 136.0, 129.7, 129.6, 119.6, 100.8, 37.1, 29.7, and 27.4.

*5-tert-Butyl-6-chloro-N-(3-iodo-4-methylphenyl)pyrazine-2-carboxamide* (**12**). Yield 82%; Anal. Calcd. for C_16_H_17_ClIN_3_O (429.7): 44.72% C, 4.99% H, 9.78% N; Found: 44.57% C, 4.67% H, 9.56% N; Mp 122.9–123.5 °C; Log *P*: 5.46; Clog *P*: 5.36969; IR: 3359, 2970, 2931, 2871, 1692, 1582, 1520, 1375, 1148, and 1060 cm^-1^; ^1^H-NMR (CDCl_3_) δ: 9.28 (1H, bs, NH), 9.25 (1H, s, H3), 8.23 (1H, d, *J* = 2.2 Hz, H2´), 7.67 (1H, dd, *J* = 8.2 Hz, *J* = 2.2 Hz, H6´), 7.24 (1H, d, *J* = 8.0 Hz, H5´), 2.42 (3H, s, CH_3_), 1.55 (9H, s, CH_3_); ^13^C-NMR (CDCl_3_) δ: 164.7, 159.7, 145.8, 140.8, 140.2, 138.0, 135.6, 129.8, 129.7, 119.7, 100.7, 39.0, 28.3, and 27.5.

### 3.2. Lipophilicity calculations

Log *P*, *i.e.*, the logarithm of the partition coefficient *P* for *n*-octanol/water, and CLog *P* values (the logarithm of *n*-octanol/water partition coefficient *P* based on established chemical interactions) were calculated using CS ChemOffice Ultra ver. 11.0 (CambridgeSoft, Cambridge, MA, USA).

### 3.3. Biological methods

Two antimycobacterial assays were used and the assays were run under different conditions. The first antituberculosis assay, provided by the Regional Hospital in Pardubice (Czech Republic), used the strains: *M. tuberculosis* H37Rv, *M. kansasii* Hauduroy CNCTC My 235/80, *M. avium* CNCTC My 80/72, and *M. avium* 152/74 (Catalogue of Strains, National Institute of Public Health, Prague, Czech Republic). The cultures were up to ten days old and the culture medium used was Šula’s semisynthetic medium (Trios, Prague, Czech Republic) at pH 5.5 and 37 °C; microdilution panel method was used. The compounds **1**-**12** were added to the medium in DMSO solution. The ability of the compounds to inhibit mycobacterial growth was determined after 7 and 14 days. PZA was used as a standard. The following concentrations were used: 128, 64, 32, 16, 8, 4, and 2 µmol/L. MIC was the lowest concentration of a substance, at which the inhibition of the growth of mycobacteria occurred [16]. For the results see Table 1. The second antituberculosis assay was performed by the TAACF screening (Tuberculosis Antimicrobial Acquisition and Coordinating Facility by The National Institute of Health of the US government). All compounds were initially screened against *M. tuberculosis* strain H37Rv in the Dose Response assay. This assay was the TAACF’s primary screen. The assay returns IC_90_, IC_50_, and all of the %Inh values at the tested concentrations. IC stands for ’inhibitory concentration’ - this was the concentration where a drug inhibits the TB strain by 90% or 50%. Compounds were considered active in the dose response screen if IC_90_ ≤ 10 µg/mL [17]. For the results see Table 1.

## 4. Conclusions

A series of twelve pyrazinamide analogues with a -CONH- linker connecting the pyrazine and benzene rings was synthesized by the condensation of chlorides of substituted pyrazinecarboxylic acids with ring-substituted anilines and characterized. The results of *in vitro* antimycobacterial screening indicated some interesting antimycobacterial activity. Three synthesized compounds (**1**, **5**, and **9**) had minimal four times higher antimycobacterial activity (MIC ≤ 2 mg/L) against *M. tuberculosis* in comparison with the standard PZA (MIC = 8 mg/L). In the second type of antituberculosis assay (TAACF), another iodo derivative 5-*tert*-butyl-6-chloro-*N*-(3-iodo-4-methyl-phenyl)pyrazine-2-carboxamide (**12**) was considered active (IC_50_ = 0.728 µg/mL, IC_90_ = 0.819 µg/mL) in comparison with PZA (IC_90_ > 20 µg/mL). Some discrepancies between both screening results might be explained by the different laboratory conditions (pH, growth medium) used. The optimal Clog *P* values (less than 3.0 or higher than 5.3) for the title compounds has been proposed.

## Figures and Tables

**Table 1:** Antimycobacterial activity (MIC, IC_90_ and IC_50_ values) of the tested compounds **1-12** in comparison with standard (PZA).

Compd.	Regional Hospital in Pardubice (Czech Republic)				TAACF (USA)	
	MIC [mg/L^-1^]				*M. tuberculosis* H37Rv	
	*M. tuberculosis* H37Rv	*M. kansasii* CNCTC My 235/80	*M. avium* CNCTC My 66/72	*M. avium* 152/74	IC_50_ [μg/mL]	IC_90_ [μg/mL]
**1**	2	128	>128	>128	>100	>100
**2**	8	32	>128	>128	>100	>100
**3**	8	>128	>128	>128	>100	>100
**4**	8	>128	>128	>128	>100	20.703
**5**	2	>128	>128	>128	>100	>100
**6**	>128	>128	>128	>128	>100	>100
**7**	32	>128	>128	>128	>100	>100
**8**	16	>128	>128	>128	>100	>100
**9**	<2	>128	>128	>128	>100	69.099
**10**	8	>128	>128	>128	>100	>100
**11**	8	>128	>128	>128	>100	>100
**12**	4	>128	>128	>128	0.728	0.819
PZA	8	>128	>128	>128	-^a^	>20^b^

^a^ not obtained; ^b^ from reference, test date 05/30/2007 [17].
